# Case of General Anasarca., with Albuminous Urine, in an Infant Ten Weeks Old

**Published:** 1848-11

**Authors:** W. Bird Herapath

**Affiliations:** Surgeon to St. Peter’s Hospital, Bristol


*Case of General Anasarca, with Albuminous Urine, in an Infant ten weeks old.* By W. Bird Herapath, M. B., Surgeon to St. Peter's Hospital, Bristol.—March 17, 1848. Mrs. Penfold brought her infant to me, complaining that it had "swollen very much all over." This state came on gradually about a fortnight ago, and she could assign no cause for its appearance. It was a male infant about ten weeks old; the whole surface had a peculiar anæmic aspect; the eyelids were transparent and swollen from œdema; the face was also somewhat anasarcous. The inferior extremities and the hands showed this condition the most; they were at least double the usual size, and pitted deeply upon pressure. The child's health did not appear to be otherwise disordered; it ate its food well, and took the breast also; it was by no means a restless child, for she had generally "very good nights" with it. The mucous membranes all appeared in good order; there was no bronchitis nor other symptoms of catarrh. It generally made plenty of water, and appeared to thrive very well until this came on. At a subsequent period I obtained a small quantity of the urine, it was clear, but high colored, and deposited abundance of albumen, with nitric acid, and heat.

These symptoms gradually became worse, in spite of all treatment; occasionally all the swelling disappeared, but it soon returned. I saw the child frequently, but never found the albumen to decrease in the urine. Its food appeared to do it no good; the nutritive function was decidedly interfered with.

About the latter end of April the infant became drowsy and comatose, and the anasarca was at this time worse than ever; convulsions ensued and rapidly carried it off.

The infant died twenty-four hours after their first appearance; it was then four months and a fortnight old.

*Post-mortem* examination, May 1st, 1848. A most careful examination was instituted. The whole thoracic and abdominal organs were perfectly healthy in structure; the kidneys had not the slightest approach to granular disease, and no structural cause was elicited for the death of the little patient. All the organs were remarkably anæmic and pale; I never saw an instance in which there was so great a deficiency of colouring matter. Had the patient been slowly bled to death, it could not have been more bloodless and pallid; the liver alone had any appearance of blood in its tissues. It was in a slight degree congested in the hepatic venous system.

*Remarks.*—The treatment consisted at first of diaphoretics. Antimony and nitric æther, combined with small doses of tinct. of digitalis, were first administered, and a warm bath every other night during five minutes. Some little benefit was derived from this mode of treatment, but eventually it was of no service. The nitro-hydrochloric acid was then tried, and the mother fancied more benefit was obtained from it than from the other attempts. This was continued for about three weeks, but after this I saw no more of the child until called upon to give a certificate of its death, when I of course demanded an examination of the body. Anæmia appears to have been the cause of the general anasarca, but the mal-assimilation of its nutriment was probably the primary cause of the albuminuria and anæmia consequent upon it.—*Prov. Med. nnd Surg. Journ.*

